# FEM Modeling of In-Plane Stress Distribution in Thick Brittle Coatings/Films on Ductile Substrates Subjected to Tensile Stress to Determine Interfacial Strength

**DOI:** 10.3390/ma11040497

**Published:** 2018-03-27

**Authors:** Kaishi Wang, Fangzhou Zhang, Rajendra K. Bordia

**Affiliations:** 1 Aerospace Research Institute of Materials & Processing Technology, Beijing 100076, China; bobwang1983@hotmail.com; 2 Research Center for Composite Materials, School of Materials Science and Engineering, Shanghai University, Shanghai 200072, China; zhangfzh@i.shu.edu.cn; 3 Department of Materials Science and Engineering, Clemson University, Clemson, SC 29634, USA

**Keywords:** polymer derived ceramic coatings, FEM modelling, thick films, in-plane Stress, interfacial shear strength, crack density

## Abstract

The ceramic-metal interface is present in various material structures and devices that are vulnerable to failures, like cracking, which are typically due to their incompatible properties, e.g., thermal expansion mismatch. In failure of these multilayer systems, interfacial shear strength is a good measure of the robustness of interfaces, especially for planar films. There is a widely-used shear lag model and method by Agrawal and Raj to analyse and measure the interfacial shear strength of thin brittle film on ductile substrates. The use of this classical model for a type of polymer derived ceramic coatings (thickness ~18 μm) on steel substrate leads to high values of interfacial shear strength. Here, we present finite element simulations for such a coating system when it is subjected to in-plane tension. Results show that the in-plane stresses in the coating are non-uniform, i.e., varying across the thickness of the film. Therefore, they do not meet one of the basic assumptions of the classical model: uniform in-plane stress. Furthermore, effects of three significant parameters, film thickness, crack spacing, and Young’s modulus, on the in-plane stress distribution have also been investigated. ‘Thickness-averaged In-plane Stress’ (TIS), a new failure criterion, is proposed for estimating the interfacial shear strength, which leads to a more realistic estimation of the tensile strength and interfacial shear strength of thick brittle films/coatings on ductile substrates.

## 1. Introduction

Ceramics, including ceramic matrix composites (CMC), are often used together with metallic components in various industrial applications. As an example, ceramic coatings made from polymer derived ceramics (PDCs) [1,2,3] have been investigated recently as a promising environmental barrier coating system at high temperatures [4,5,6,7,8,9]. The interface, which was created by the direct bonding between the ceramic and metallic substrate, is a critical element and potential failure location in such multilayer structures. The substantially different properties of these two classes of materials, especially the thermal expansion coefficient and modulus of elasticity, lead to the formation of unrelaxed stresses during service that may induce failures that were initiated at the interface. Therefore, it is important to measure and understand the failure mechanism of such interfaces so that coating material’s processing and structure can be optimized. 

Cracking in layered structures and failure mechanism of different interfaces have been extensively studied in the mechanics community [10,11,12,13]. Particularly, the interfacial properties of coatings/films on metallic or ceramic substrates draw wide attention due to its engineering importance. Approaches that are taken for this topic are mainly focused on energy (energy release rate) and stress (induced decohesion/delamination) perspectives [14,15,16,17]. Some of the research employed finite element method (FEM) to simulate the stress/strain states around the failure area [18,19,20], but interfacial properties exhibit non-negligible dependency on intrinsic factors of the studied material itself, such as defects, flaws, and residual stress [21,22]. In addition, many techniques have also been developed to experimentally measure interfacial properties (e.g., adhesion strength) of different materials and structures, such as indentation [23], peeling [24], tension/shear [22,25], scratch [26], bending [27], and tearing-off tests [28]. However, specific limitations that are associated with these techniques limit their use, including weak glue strength (peeling, bending, and tearing-off tests), complex configuration (scratch test), and additional in-plane compressive loading (indentation test).

Agrawal and Raj (A-R) [29,30] developed an experimental technique and analysis to evaluate the ultimate shear strength of a ceramic-metal interface. Due to its simplicity in experimental setup and its acceptable reliability in property prediction, the A-R model has been widely used in many reported works [31,32,33,34,35,36,37]. Furthermore, Chen et al. [21] reported their modification of the sinusoidal profile of interfacial shear stress in the original A-R model and test results on a TiN (coating)—304 steel (substrate) system. It is noteworthy to point out that, besides films below or around 1 µm thick [19,21,33,36,37,38], coatings of tens or even hundreds of micron thick have also been reported to be tested using the original A-R model [31,32,39]. In these experimental papers, defects and residual stress are primarily mentioned and discussed as factors that are leading to the error in interfacial property prediction, nevertheless, thickness and in-plane stress distribution have been largely overlooked as a potential source of error.

**Figure 1:**
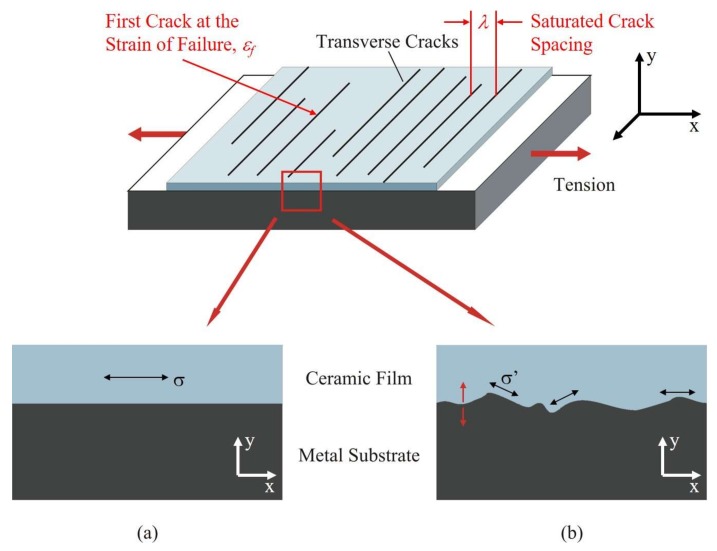
The geometry of the test and the definition of the various quantities with (**a**) referring to the stress state when the interface is flat and (**b**) when the interface is rough. *x*-axis: direction of applied tension; *y*-axis: direction perpendicular to the plane of the coating.

In A-R’s experiments, a thin film (nanometer scale) of silica ceramic was deposited onto two types of metallic substrates: copper and nickel. During deformation of the metal in tension, shear stress is generated at the interface and normal tensile stress is transferred into the film. As the tensile strain in the metal increases during testing, cracks transverse to the tension direction start to appear in the ceramic film. The strain in the substrate at the first appearance of the transverse cracks is related to the tensile fracture strength of the film. As the strain that is applied to the metal substrate increases, the crack density increases until it reaches a saturation value (Figure 1). The crack spacing at the saturated crack density is related to the interfacial shear strength of the ceramic-metal interface. The key finding lies in the relationship between the interfacial shear strength ($\hat{\tau }$), film thickness (δ), average saturated crack spacing (λ), maximum crack spacing at saturation ($\lambda_{\max}=\;1.5\lambda$), the applied tensile stress (σ), and the tensile strength of the ceramic film ($\hat{\sigma }$), as shown below [29]:

(1) $$\hat{\tau }=\frac{\pi \delta }{\lambda_{\max}}\hat{\sigma }\;\mathrm{or}\;\hat{\tau }=\frac{\pi }{1.5}\frac{\delta }{\lambda }\hat{\sigma }$$

(2) $$\hat{\sigma }=\varepsilon_{f}E$$

where *ε_f_* and *E* are the fracture strain and Young’s modulus of the ceramic film, respectively. *ε_f_* is also the strain in the metal substrate at the point of formation of the first crack in the film, since it is assumed that the interface is perfectly bonded with no slip. Following this approach, Agrawal and Raj determined the shear strengths of copper-silica (78 nm thick) and nickel-silica (42 nm thick) interfaces to be 0.9 and 1.4 GPa. The authors also claimed that the margin of error in this technique is estimated to be less than 20%. A detailed description of the theory can be found in their original papers [29,30]. Here, we would like to point out three critical assumptions that were made in this model: films are sufficiently thin, there is no slip at the interface and the in-plane stress in the film is uniform in the thickness direction.

Torrey et al. [40] carried out this experiment with a thick PDC coating (~18 μm) on a 316 stainless steel substrate. The PDC coating was composed of poly (hydridomethylsiloxane), as the precursor of SiOC ceramic and matrix, and TiSi_2_ as an active filler. The prepared slurry was dip-coated onto the substrate with 600-grit surface finish. Then, the coated substrate was heat treated at 150 °C (crosslinking) and 800 °C (pyrolysis) for 2 h, respectively. The geometry of the interfacial strain samples is 30 mm × 10 mm × 2 mm. During the tension test, substrates were loaded at a constant crosshead velocity of 0.5 mm/min (Universal Testing Machine, Instron 4505) to the desired strain state, such as 5, 7.5, 10, 15, 20%. Data, regarding fracture strain, crack spacing, etc., were collected by graphic observation of the samples’ microstructure. Using the experimental results and Equations (1) and (2), they determined the tensile strength of the film and the interfacial shear strength to be 3.75 and 4.7 GPa, respectively. Please refer to [40] for additional details. However, both of these values are unrealistically high for this coating system. It is postulated that the primary reason for the overestimation is non-uniform stresses in these thick films. 

To investigate this non-uniformity comprehensively and quantitatively, finite element modeling (FEM) using ABAQUS software was carried out and the results are reported in this work. The experimental results reported by Torrey [40,41] are used as the starting point and extended by conducting parametric study of the effect of important variables on the stresses and their distribution. The following sections focus on the setup of the ceramic-metal interface model used for computational simulations, the simulation results, and a possible approach to correctly estimate the in-plane stress that should be used as part of the fracture criteria for thick films. However, possible errors that are associated with the measurement of fracture strain of the coating and its effect on the interfacial shear strength are not within the scope of this paper, but will be briefly discussed.

## 2. Results

Details regarding the setup of the FEM model in ABAQUS software are presented later in Section 4: Materials and Methods, which should be referred to for a more comprehensive understanding of the following simulated results. 

**Figure 2:**
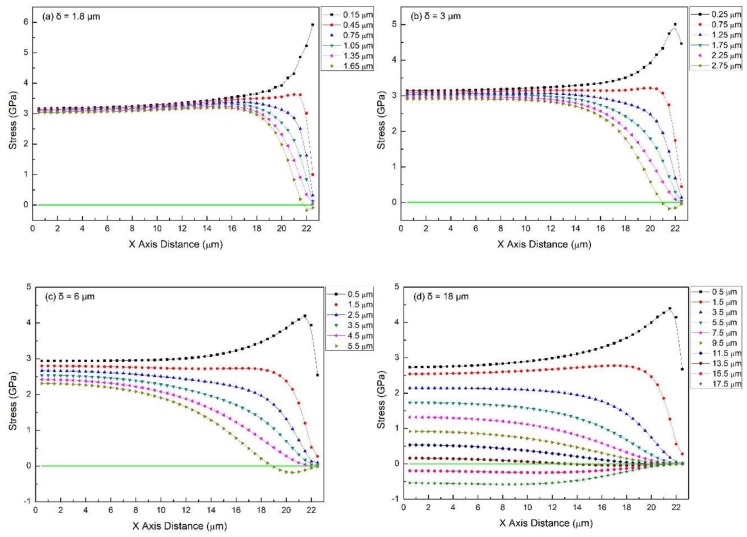
The in-plane stress distribution (dot-line plot) in films of different thicknesses for the case of the film/substrate system being tested in tension: (**a**) *δ* = 1.8 μm, (**b**) *δ* = 3 μm, (**c**) *δ* = 6 μm, (**d**) *δ* = 18 μm. Common features: *E_SS_* = 200 GPa, *E_PDC_* = 50 GPa, *λ*_max_ = 45 μm, *ε_f_* = 7.5%.

**Figure 3:**
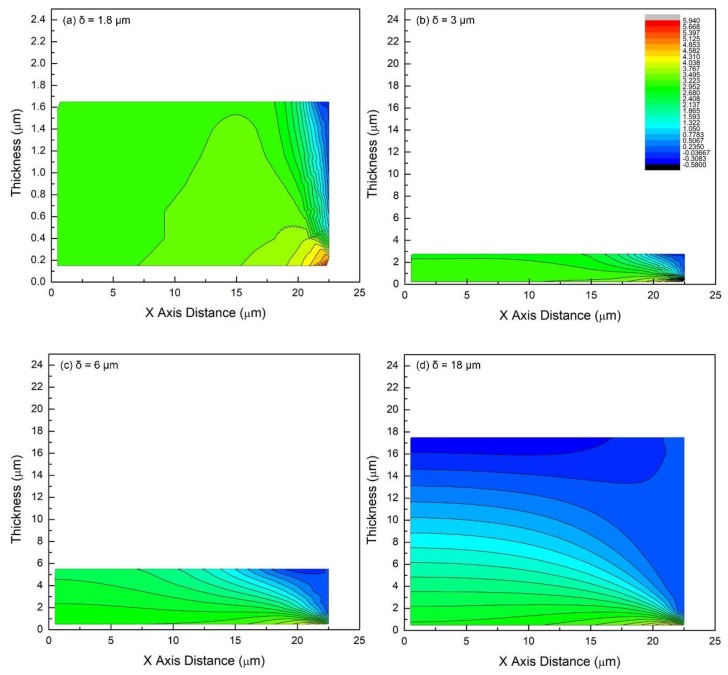
The in-plane stress distribution (two-dimensional (2D)-contour plot) in films of different thicknesses for the case of the film/substrate system being tested in tension: (**a**) *δ* = 1.8 μm, (**b**) *δ* = 3 μm, (**c**) *δ* = 6 μm, (**d**) *δ* = 18 μm. Common features: *E_SS_* = 200 GPa, *E_PDC_* = 50 GPa, *λ*_max_ = 45 μm, *ε_f_* = 7.5%. Note that the y-axis scale is different for the thinnest coating to clearly illustrate the difference in the stress distribution of thin coatings as compared to thick ones.

Once the FEM model is set up (Section 4), several key parameters can be assigned with real values for both substrate and film layers, including the Young’s moduli of both materials, film thickness, and crack spacing. To start off, parameters in Torrey’s work [41] are considered: Elastic modulus of the stainless steel substrate, *E_SS_* = 200 GPa, elastic modulus of the coating, *E_PDC_* = 50 GPa, *δ* = 18 μm, *λ*_max_ = 45 μm, *ε_f_* = 7.5%. The simulated in-plane stress distribution in the film is shown in Figure 2 and Figure 3 (same data, two ways of plotting). In the dot-line plot of Figure 2, each dot represents the stress value at a certain position in the film, i.e., distances away from the symmetric boundary (*x*-axis value) and the interface (profile curves for different planes; dots in the same stress plane, which is parallel to the interface, connect with each other to form one of the profile curves). In addition, the two-dimensional (2D)-contour plot of Figure 3 uses color scale to further visualize the stress distribution in the specific film in two dimension (red end: positive, tension; blue end: negative, compression). In these curves, the *x*-axis represents half of the saturation distance (0.5 *λ*_max_ = 22.5 μm) between the transverse cracks.

It is clear that the in-plane stress is not uniform in all of the films. The non-uniformity increases as film thickness increases and is severe for 6 and 18 μm films (Figure 2c,d and Figure 3c,d). Moreover, the stress does not decrease linearly from the interface to the free top surface as was assumed by Torrey et al. [40]. Instead, the in-plane stress decreases from both the interface and boundary planes towards the free surfaces (top surface and crack surface): a trend of diagonal nonlinear decrease. Figure 2d shows that a significant portion of the stress near top surface, e.g., dot-line of *y* = 15.5 and 17.5 μm, drops to negative values so that it forms a region where the compressive stresses are generated. Meanwhile, although very confined, there is a region of localized stress concentration near the crack edge, but close to the interface (*x* = 21.5 μm, *y* = 0.5 μm). It implies that there might be two competing failure mechanisms in the 18 μm-thick film: if crack initiates somewhere in the film body and propagate through the thickness direction, it forms transverse cracks analyzed in the Agrawal-Raj model. However, if it initiates from the stress concentration region, i.e., coordinates (21.5, 0.5), then it will propagate along the interface, and may or may not kink out of the interface as described in mixed mode fracture mechanics models [42,43]. After kinking, these interfacial cracks would propagate into one of the two materials—most likely the PDC film/coating and eventually lead to the delamination and spallation of the coating. Which one of the two possibilities occurs first is dependent on where the critical flaws/defects are. Once the strain energy is released through the formation of a new crack between two existing cracks, higher strains, resulting in higher tensile stresses, will be required to build up the strain energy for subsequent cracking. As the strain increases, it is possible to overcome the critical values for both types of cracks, and it is possible that both types of cracks may initiate and propagate simultaneously at multiple places in the film. This will result in massive film delamination, which has been observed in some cases. Nevertheless, further experimental research is needed to show experimental evidence of this prediction.

From these results, noting that the in-plane stress distribution is not uniform in thick films, a systematic parametric study can be carried out to evaluate the effects of a single parameter while fixing others.

### 2.1. Effect of Film Thickness

The conditions for this set of simulations are *E_SS_* = 200 GPa, *E_PDC_* = 50 GPa, *λ*_max_ = 45 μm, *ε_f_* = 7.5%. Film thickness is varied: 1.8, 3, 6, and 18 μm. Simulation results are summarized in Figure 2 and Figure 3.

As film thickness decreases from 18 to 3 μm, stresses in different stress planes (see graph legends of Figure 2) converge for a large part of the film and exhibit a narrower and narrower distribution. They tend to become uniform not only in the thickness direction, but also along the x-axis, namely the direction of applied stress. This tendency of stress uniformity in the film body might lead to higher unpredictability about the location of the next crack initiation. Divergent stresses are pushed toward the crack surface. The compression region in films has gradually shrunk to the upper-right corner of ‘free surface’. As indicated by the deep blue region in 2D-contour plots, it takes about 20 vol % of the film body when *δ* = 18 μm, while roughly 5 vol % or less when *δ* = 3 μm. Stress in the concentration region (21.5, 0.15~0.5; black dot-line) increases in magnitude, 4.20 to 5.92 GPa, as the thickness decreases and it is noticeably shifted toward the very edge of the crack surface. In addition, this stress concentration remains localized as it can only be observed in the stress plane closest to the interface. In-plane stress drops sharply, regardless of film thickness. Therefore, mode I delamination at the interface near crack surface could possibly be suppressed to certain degree due to the co-existence of stress concentration and compression around that region. When the film thickness is further reduced to 1.8 μm, which is one order smaller than 18 μm (Figure 2a and Figure 3a), stresses of different stress planes further converge in the most part of the film and only diverge in the vicinity of the free crack surface. At this thickness, the film appears to be more rigid under tension, and thus compression in the film becomes nearly negligible. If film thickness keeps decreasing to the nanometer scale, it can be foreseen that the in-plane stress will be uniform enough to completely obey the Agarwal and Raj model’s assumptions (uniform in-plane tensile stress) [29].

This set of simulations provides insight into the relationship between film thickness and in-plane stress distribution. Films of nanometer thick are believed to behave ‘rigidly’ under tension, no significant shear sliding between stress planes in the ‘thin’ film could take place, so that in-plane stress remain uniform throughout the thickness except the limited portion of film around free crack surface. As the film gets thicker, in-plane stress becomes non-uniform within the film. In fact, the transition between thin and thick is continuous and dependent on the coating’s and substrate’s physical properties. Thus, it could only be examined for each specific system. However, there is an upper bound of thickness with ‘nearly’ uniform in-plane stress, which we believe is still a close enough approximation to directly apply the original model. Here, we propose that for this test, if the deviation between the highest and the lowest in-plane stresses is less than 5% at *x* = 0 (central region of two adjacent transverse cracks), then the film is considered ‘thin’. If it is greater than 5%, then it is called ‘thick’. Using Equation (1), we can write this criterion for this film as:

(3) $$\frac{\tau_{2}-\tau_{1}}{\tau_{1}}=\frac{\pi \delta }{\lambda_{\max}}\cdot \left(\frac{\sigma_{2}-\sigma_{1}}{\sigma_{1}}\right)<5\%$$

Namely,

(4) $$\left|\frac{\sigma_{2}}{\sigma_{1}}\right|<\frac{\lambda_{\max}}{20\pi \delta }+1$$

Substituting *λ*_max_ = 45 μm, the upper and the lower bounds of the in-plane stresses at *x* = 0 for thickness *δ* = 1.8, 3, 6, and 18 μm in Equation (4), simulated coatings of different thicknesses can be categorized, as shown in Table 1.

For the parameters that were used in this study, this critical thickness is ~4.5 μm. When the film is much thicker, e.g., 18 μm, the in-plane stress distribution becomes complex. The top surface of the film experiences a substantial transition from tension to compression. Only the part of the film that is closest to the interface is under the same strain as the substrate, and the strain gradually reduces and even becomes compressive near the top surface. Therefore, crack propagation would be much more complex in thick films.

### 2.2. Effect of Crack Spacing for Thick Films

**Figure 4:**
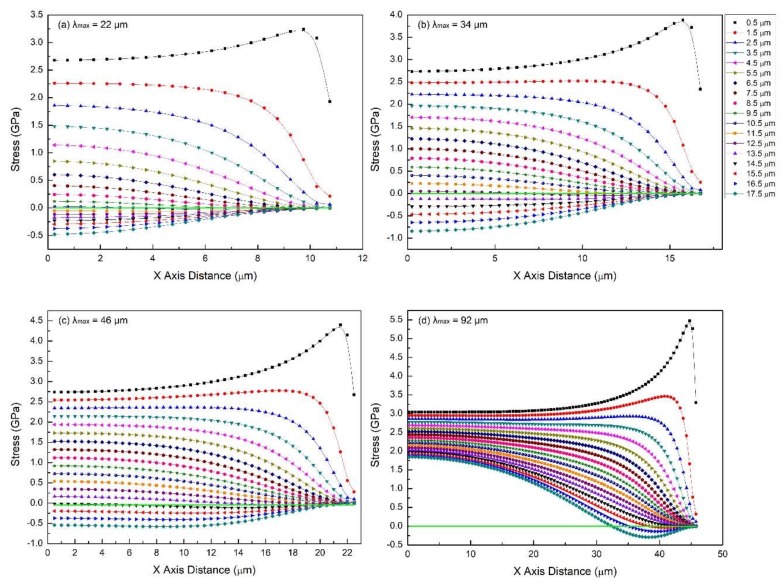
The in-plane stress distribution (dot-line plot) in films of different crack spacings for the case of the film/substrate system being tested in tension: (**a**) *λ*_max_ = 22 μm, (**b**) *λ_max_* = 34 μm, (**c**) *λ*_max_ = 45 μm, (**d**) *λ*_max_ = 92 μm. Common features: *E_SS_* = 200 GPa, *E_PDC_* = 50 GPa, *δ* = 18 μm, *ε_f_* = 7.5%.

**Figure 5:**
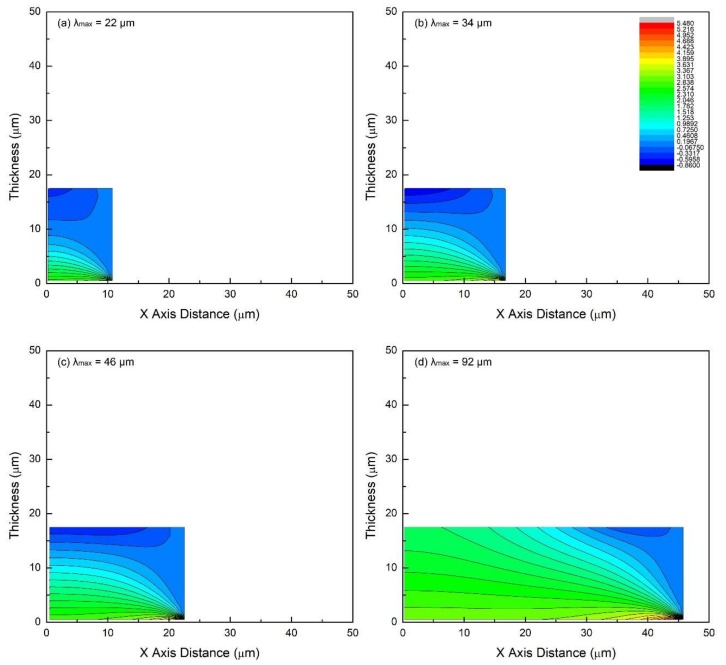
The in-plane stress distribution (2D-contour plot) in films of different crack spacing for the case of the film/substrate system being tested in tension: (**a**) *λ*_max_ = 22 μm, (**b**) *λ*_max_ = 34 μm, (**c**) *λ*_max_ = 45 μm, (**d**) *λ*_max_ = 92 μm. Common features: *E_SS_* = 200 GPa, *E_PDC_* = 50 GPa, *δ* = 18 μm, *ε_f_* = 7.5%.

The conditions for this set of simulations are *E_SS_* = 200 GPa, *E_PDC_* = 50 GPa, *δ* = 18 μm, *ε_f_* = 7.5%. Crack spacing is varied using the following values: 22, 34, 45, and 92 μm. This set of simulations is useful to understand the progressive increase in the density of transverse cracks (which results in decreasing crack spacing). Simulation results are presented in Figure 4 (dot-line) and Figure 5 (color contour).

From the previous section, it is known that for thick films (*δ* = 18 μm), in-plane stress is strongly dependent on the distance from the interface. However, its distribution is found to evolve with the process of the tension test as well. The crack spacings presented here—from 92 to 22 μm—are a reflection of increasing crack density to saturation. So, when the crack spacing is large, e.g., *λ*_max_ = 92 μm (Figure 4d and Figure 5d), the cracks are far apart. The region in the film body, which is affected by the compressive strain due to the free crack surface, becomes quite limited, as seen in Figure 5d (deep blue region). In-plane stress distribution in the major part of the film tends to be narrow. In fact, if the crack spacing is infinite, film would behave as if there were no cracks at all, resulting in nearly the same stress distribution through the thickness. 

Although *λ*_max_ = 92 μm is a large crack spacing, the top surface is partially in compression. As the tension test proceeds, more and more of the film goes into compression as crack spacing gets shorter (*λ*_max_ = 45 to 22 μm). The peak stress in the stress concentrating region with coordinates near (9.75~21.5, 0.5) decreases from 4.4 to 3.24 GPa, however, the upper- and lower-bounds of the magnitude of in-plane stress does not vary significantly. 

From these results, it is clear that for these thick coatings, soon after the first few cracks are formed, the stress state undergoes a significant transition. Specifically, an increasing volume of the coating transitions from tensile to compressive stresses. Thus, in-plane stress distribution in thick films is non-uniform almost for the entire process.

### 2.3. Effect of Film Modulus for Thin Films

**Figure 6:**
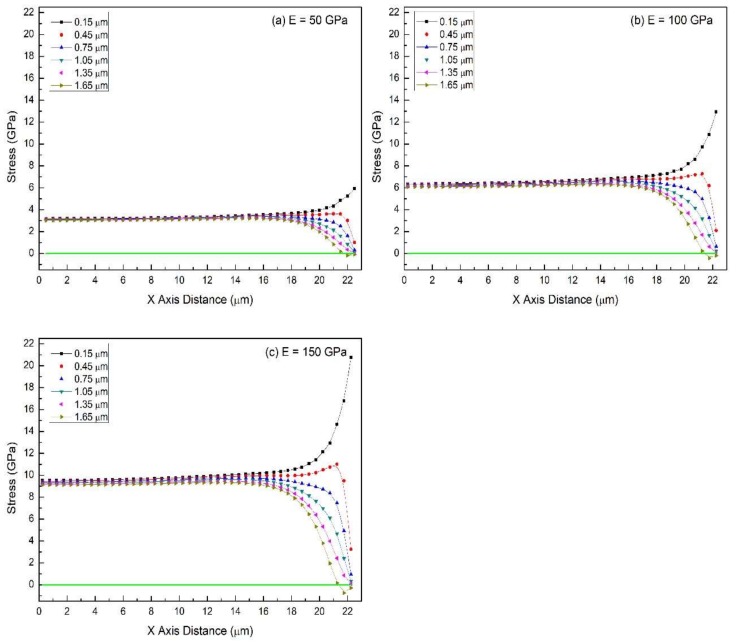
The in-plane stress distribution (dot-line plot) in films of different Young’s modulus for the case of the film/substrate system being tested in tension: (**a**) *E* = 50 GPa, (**b**) *E* = 100 GPa, (**c**) *E* = 150 GPa. Common features: *E_SS_* = 200 GPa, *δ* = 1.8 μm, *λ*_max_ = 45 μm, *ε_f_* = 7.5%.

**Figure 7:**
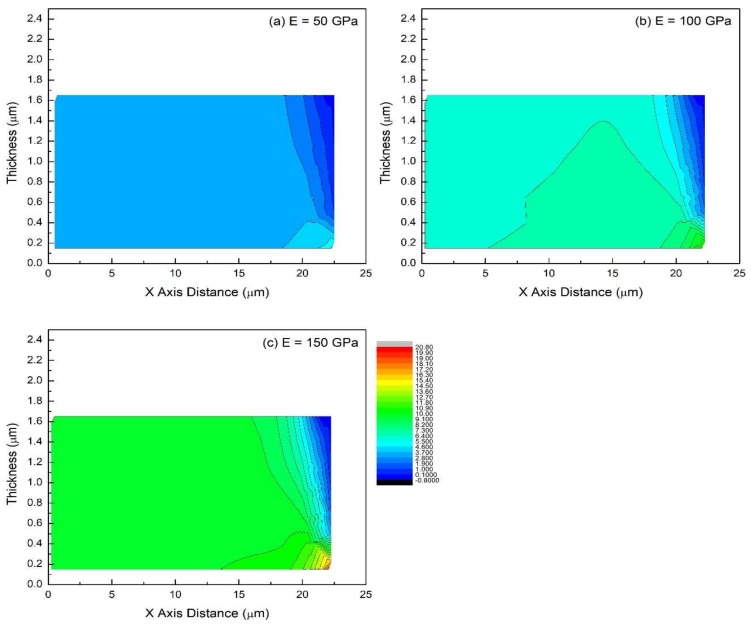
The in-plane stress distribution (2D-contour plot) in films of different Young’s modulus for the case of the film/substrate system being tested in tension: (**a**) *E* = 50 GPa, (**b**) *E* = 100 GPa, (**c**) *E* = 150 GPa. Common features: *E_SS_* = 200 GPa, *δ* = 1.8 μm, *λ*_max_ = 45 μm, *ε_f_* = 7.5%.

The conditions for this set of simulations are *E_SS_* = 200 GPa, *δ* = 1.8 μm, *λ*_max_ = 45 μm, *ε_f_* = 7.5%. Film’s Young’s modulus is varied with values equal to 50, 100 and 150 GPa. Simulation results are shown in Figure 6 and Figure 7. The smallest value of film thickness was chosen to explore whether increasing the film modulus has any effect on the stress distribution for this case in which the stress is approximately uniform for low modulus films (50 GPa). 

Regardless of the films elastic modulus, the 1.8 μm-thick films exhibit nearly uniform in-plane stress distribution in the major part of the film body. At the same crack spacing, the in-plane stress increases proportionally as the film gets stiffer (higher modulus). For example, average stress at *x* = 0 is ~3.10, ~6.21, and ~9.31 GPa when *E* = 50, 100, and 150 GPa, respectively (Figure 6). In addition, maximum stress in the stress plane adjacent to the interface (*y* = 0.15 μm) shows a similar trend, rising from 5.92 to 20.75 GPa—a 3.5 time increase. The 2D contour plots (Figure 7) visually reveal their comparable stress patterns, except for different overall stress levels/colors. Therefore, when the film is thin enough, the in-plane stress distribution pattern is insensitive to the changes in the film’s Young’s modulus. The stiffer the film is, the higher the stress is at a given strain as expected.

## 3. Discussion

For the test developed by Agrawal and Raj to measure the interfacial strength of metal/ceramic interfaces [29,30], the effects of film thickness, crack spacing, and film Young’s modulus on the in-plane stress distribution in the PDC coating/film have been investigated using the finite element method. The major conclusions are: first, film thickness determines whether the in-plane stress distribution is uniform in the thickness direction or not (at any given *x* value away and from the free surfaces or a crack)—the thinner the film, the more uniform the stress. Second, crack spacing governs the value of the overall tensile stress in the film at certain strain—the smaller the crack spacing, the larger the compressive region, and thus the smaller the overall stress. Finally, the film’s stresses scale with the elastic modulus of the film at a given strain—the higher the modulus, the higher the stresses.

Typical PDC coatings are in the thick film regime with a thickness in the range of tens to hundreds of microns. The simulations show that assumption of constant in-plane tensile stress, in the test to measure the interfacial shear strength, is not valid. The highest stresses are always at the interface and therefore the assumption of uniform stress would overestimate the strength of the coating and the interfacial shear strength since transverse cracks would originate from pre-existing flaws and not necessarily from the interface. Furthermore, the stress patterns exhibit the non-linear characteristic in all of the simulated cases here. As a result, the assumption of linear stress from the interface to free surface and use of average stress (half of the peak stress) to estimate the interfacial strength is also not valid. A more realistic single valued stress is needed for use in Equations (1) and (2) of the classical model for thick films. 

**Figure 8:**
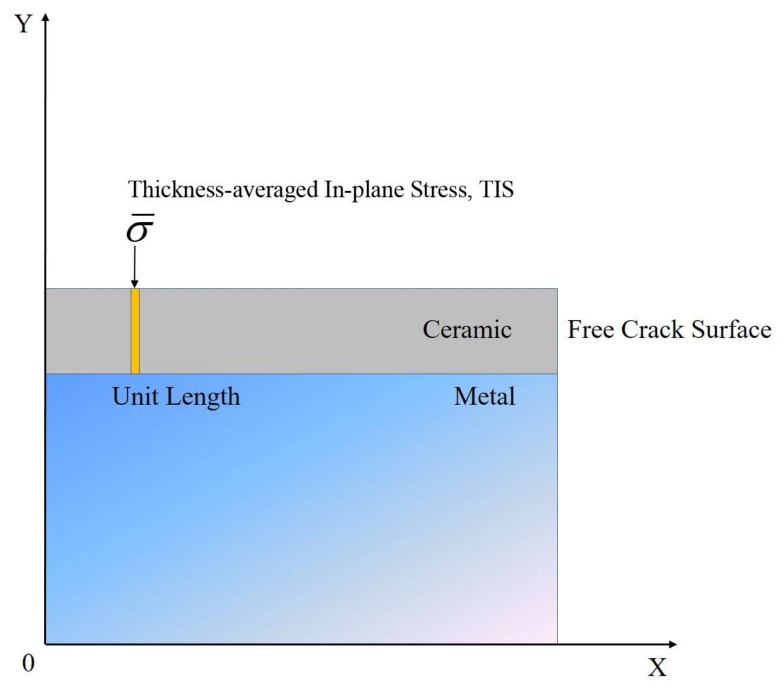
Schematic of the ‘Thickness-averaged In-plane Stress’ (TIS) in the film at a given *x* value.

We propose ‘Thickness-averaged In-plane Stress’ (TIS), defined as the thickness averaged value of stress at a given *x* value, to be the single valued stress for thick films (Figure 8). It takes into account the sign of the stress (tension at the interface and the possible compression near free surface). The TIS is postulated to be the driving force for cracking since the transverse cracking is governed by both a volume average of stresses and the need for tensile stresses. The margin of errors in determining thick film’s tensile and interfacial shear strengths is expected to be reduced by applying TIS into the classical model instead of other simple assumptions (e.g., a linear distribution of stresses through the thickness). Using the stress distributions that are plotted in Figure 2, Figure 3, Figure 4, Figure 5, Figure 6 and Figure 7, this stress can be calculated as a function of distance from a free edge (or crack).

**Figure 9:**
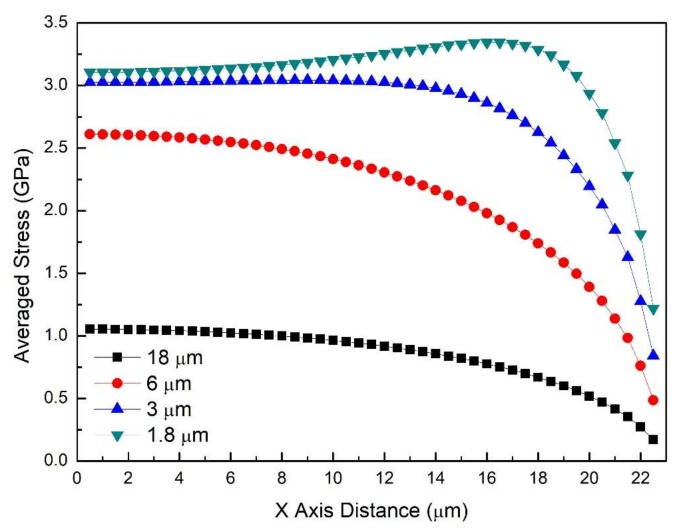
The TIS distribution in films of 1.8, 3, 6, and 18 μm thick.

Figure 9 illustrates the TIS as a function of the distance from the crack surface for films of different thicknesses. All of the films possess the same Young’s modulus (50 GPa), crack spacing (45 μm), and strain (7.5%). It can be seen that at any value of *x*, the TIS increases as the film gets thinner. The maximum of each TIS curve can be observed at *x* = 0, the midpoint between two transverse cracks, except for the 1.8 μm thick film that shows a slight stress plateau before the compression region. The minimum is always at the crack surface, implying the stress concentration near the interface in this region is largely suppressed. The overall stress profile increases a significant amount (3-fold increase from 1.05 to 3.03 GPa at *x* = 0) when the thickness is reduced from 18 to 3 μm, however, it is only 3.11 GPa when it was further reduced to 1.8 μm. Note that both 1.8 and 3 μm thick films show a similar characteristic of converging in-plane stresses in Figure 2a,b, so that their TIS is constant for most part of the film, while thicker films, i.e., 6 and 18 μm, show decreasing TIS (Figure 9).

**Figure 10:**
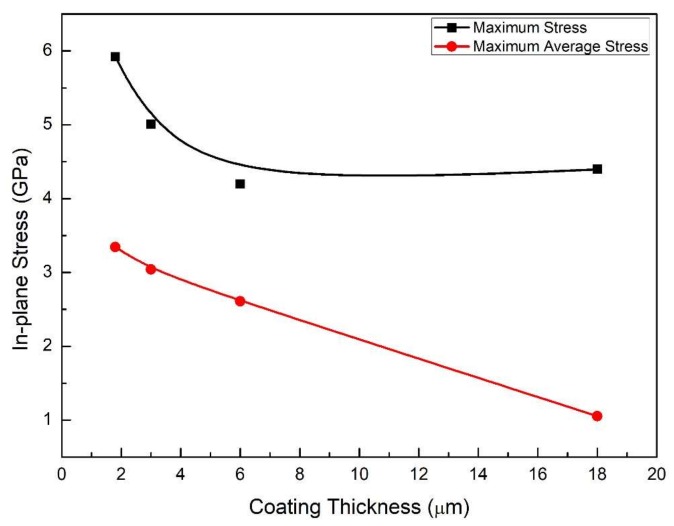
A comparison of the maximum TIS (red) and the maximum in-plane stress (black) found in the films of a series of thicknesses.

Therefore, when the thick film is under tension, it is more likely to crack at or around the midpoint between two existing cracks, where there is higher TIS. Materials defects might introduce certain randomness in the exact position of the ‘next crack’. Further, TIS is significantly (a factor of 2 to 5) lower than the maximum stress that is found in the films. The ratio of the TIS to maximum stress decreases as film thickness increases (Figure 10): the TIS decreases almost linearly as a function of the thickness, while the maximum stress reaches a plateau for film thickness of more than 6 μm.

**Figure 11:**
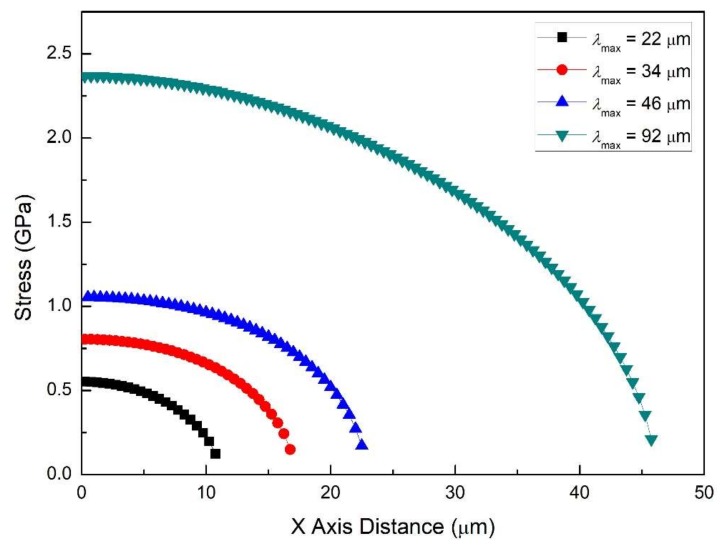
The TIS distribution for several crack spacing. The TIS, at any location, decreases as the crack spacing decreases.

As the tension test proceeds with increasing strain, strain energy is stored in the coating. Formation of transverse cracks is one of the important ways this strain energy is released. As the strain in the substrate increases, more and more cracks form until reaching saturated crack density. As the crack density increases, crack spacing decreases. The TIS curves for a series of crack spacings (Figure 11) reveal that the overall stress level goes down as the crack spacing decreases. When the TIS becomes lower than the tensile strength of the film, no additional cracks are formed and saturation crack density is achieved. It should be noted that Figure 11 is for one value of the film strain. Additional simulations are needed for the dynamic case in which both the crack spacing decreases and the film strain increases.

Finally, we apply these simulations and the TIS to a PDC coating system that was pyrolyzed at 800 °C with the following properties: *E_SS_* = 200 GPa, *E_PDC_* = 50 GPa, *δ* = 18 μm, *λ*_max_ = 45 μm, *ε_f_* = 7.5%, and saturation strain *ε_s_* = 10% [40]. In [40], assuming constant in-plane stresses, the tensile strength of the coating was calculated to be 3.75 GPa and the interfacial strength 4.7 GPa. Assuming a linear variation in in-plane stresses, the tensile strength and the interfacial shear strength were calculated to be 1.9 GPa, and 2.35 GPa, respectively. However, Figure 9 shows that the maximum TIS found in the 18 μm thick coating is just 1.05 GPa, which is essentially the tensile strength (*σ_ts_* or $\hat{\sigma }$). Further substituting the simulated TIS result (1.05 GPa) into Equation (1), the interfacial shear strength can be obtained: $\hat{\tau }$ = 1.32 GPa. These results are only 28% of those calculated using the constant stress assumption of thin films—a significant reduction due to realistic simulations of the stress distribution. 

It is noteworthy to point out that the tensile strength of 1.05 GPa is higher than the expected strength for such PDC coatings. The most likely reason for this is the difficulty in measuring the strain at which the first crack forms. From Equation (2) and the results of this paper, the tensile strength of the films is equal to the TIS, which is proportional to the strain at which the first crack is observed. The reported value of *ε_f_* = 7.5% in [40] is too high as failure strain for ceramics. In addition to the experimental difficulty of identifying the first crack, due to the stress distribution decreasing as the distance from the interface increases, it is clear that the cracks at the interface form at a much lower strain than the strain at which these are observed on the top surface. Approaches to address both of these issues are currently under investigation. 

## 4. Materials and Methods: FEM Modelling 

**Figure 12:**
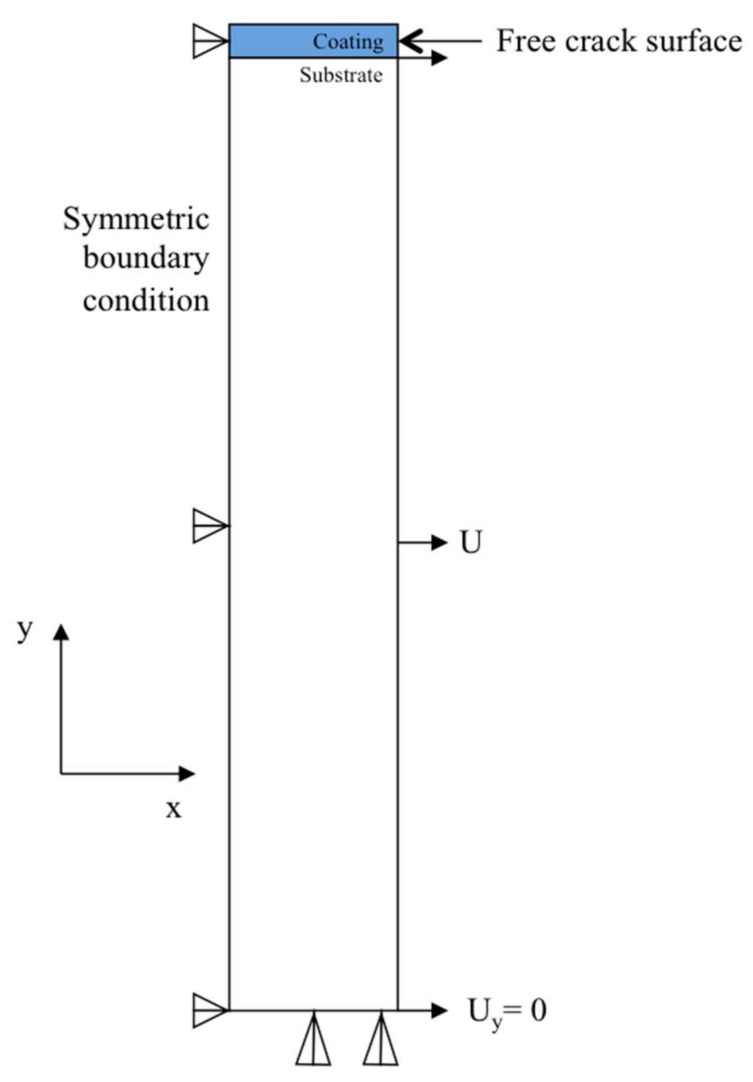
Schematic of the unit cell in finite element method (FEM) model: the cross-sectional geometry of the ceramic-metal interface. *x*-axis: direction of applied tension; *y*-axis: direction perpendicular to the plane of the coating.

Cracks in a coating/film layer are assumed to be periodic. A representative unit cell is taken out and studied. Because of the geometric symmetry about *y*-axis, only half of the unit cell is modeled, as shown in Figure 12. The blue part on top represents the coating layer in this study, and beneath it is the metallic substrate. The simulated lengths of the coating segments are 22, 34, 45, and 92 μm, and the thicknesses are 1.8, 3, 6, and 18 μm, respectively, depending on the different studied scenarios. The thickness of the substrate is 45 μm. This unit cell is to scale along the *y*-axis. The width of the unit cell along the *x*-axis is half of the saturation crack spacing. Bottom of the substrate is fixed and the top surface of the coating is constraint-free. Symmetric boundary condition is applied on the left side of the unit cell, so that only one crack surface needs to be studied in the simulation and the other is considered to be identical but in the opposite direction. The right side of the coating is a free edge to represent the constraint-free state of the crack surface. Tensile strain of 7.5% is imposed only on the right side of substrate in the *x*-direction. As a consequence, the stresses from the substrate are transferred to the coating by shear stresses at the interface. No slip (or sliding) is assumed at the interface. The properties of the coating and the substrate are assumed to be linear elastic. Young’s modulus of the PDC coating is 50 GPa, while that of the substrate is 200 GPa. The model is meshed with 8-node biquadratic quadrilateral elements and is assumed to be 2D planar strain. The approximate global size of element is 1. However, the mesh size along the coating thickness is 0.3. As it is a static problem, the time step and the increments of time are set to be 1. The simulations were conducted using ABAQUS 6.14-2. Effects of several key factors, such as crack spacing, coating thickness, and elastic modulus on the stress distribution in the coatings were studied by running numerical simulations under different conditions.

## 5. Conclusions

This work has used a finite element method to study the non-uniform in-plane stress distribution in thick PDC coatings on metallic substrates in which the tensile stresses are applied on the substrate. The effects of three key parameters—film thickness, crack spacing, and Young’s modulus of the film—are independently investigated. It is found that the in-plane stress has non-linear gradient in both in-plane and thickness directions. However, if the film is sufficiently thin, the stress across the thickness at any cross-section converges and becomes uniform, except at the free surfaces. Crack spacing and Young’s modulus predominantly effect the magnitude of the stress.

The classical model of Agrawal and Raj, originally proposed and used for thin films, is adapted by introducing the ‘Thickness-averaged In-plane Stress’ (TIS) to account for the stress gradient in the thickness direction of thick films. Use of this results in values for tensile strength and interfacial shear strength that are about 30% of those that were obtained using constant in-plane stress. Other reasons contributing to the higher than expected fracture strength and interfacial shear strength are also discussed. These include accurate measurement of the strain at the point of first cracking at the interface. An additional factor that must be considered is the interaction between the transverse cracks and possible film delamination due to stress concentration. Further numerical simulations and experiments are required to properly account for these factors.

## Figures and Tables

**Table 1:** Categorization of coatings with different thicknesses into ‘thin’ and ‘thick’.

Coating Thickness (µm)	$\left|\frac{\sigma_{2}}{\sigma_{1}}\right|$	$\frac{\lambda_{\max}}{20\pi \delta }+1$	Equation (4) Holds?	‘Thin‘ or ‘Thick‘
1.8	1.04	1.40	Yes	Thin
3	1.08	1.24	Yes	Thin
6	1.27	1.12	No	Thick
18	5.05	1.04	No	Thick
